# Collagen Remodeling of Strattice™ Firm in a Nonhuman Primate Model of Abdominal Wall Repair

**DOI:** 10.3390/bioengineering12080796

**Published:** 2025-07-24

**Authors:** Kelly Bolden, Jared Lombardi, Nimesh Kabaria, Eric Stec, Maryellen Gardocki-Sandor

**Affiliations:** 1 Cultura Dermatology and Plastic Surgery, Washington, DC 20015, USA; 2 Department of Surgery, Howard University, Washington, DC 20059, USA; 3 Allergan Aesthetics, an AbbVie Company, Branchburg, NJ 08876, USA

**Keywords:** surgical meshes, hernia, acellular dermal matrix, collagen, inflammation

## Abstract

This study characterized collagen remodeling in an electron-beam-sterilized porcine acellular dermal matrix (E-PADM) by evaluating host response kinetics during wound healing. E-PADM (*n* = 6 lots/time point) was implanted in an abdominal wall bridging defect in nonhuman primates (*N* = 24). Histological, immunohistochemical, and biochemical assessments were conducted. Pro-inflammatory tissue cytokines peaked 1 month post-implantation and subsided to baseline by 6 months. E-PADM-specific serum immunoglobulin G antibodies increased by 213-fold from baseline at 1 month, then decreased to <10-fold by 6–9 months. The mean percentage tissue area staining positively for matrix metalloproteinase-1 plateaued at 3 months (40.3 ± 16.9%), then subsided by 6 months (16.3 ± 11.1%); tissue inhibitor matrix metalloproteinase-1 content plateaued at 1 month (39.0 ± 14.3%), then subsided by 9 months (13.0 ± 8.8%). Mean E-PADM thickness (1.7 ± 0.2 mm pre-implant) increased at 3 months (2.9 ± 1.5 mm), then decreased by 9 months (1.9 ± 1.1; equivalent to pre-implant). Histology demonstrated mild inflammation between 1–3 months, then a peak in host tissue deposition, with ≈75%–100% E-PADM collagen turnover, and fibroblast infiltration and neovascularization between 3–6 months. Picrosirius red staining revealed that mature E-PADM collagen was replaced by host-associated neo-collagen by 6 months. E-PADM implantation induced wound healing, which drove dermal E-PADM collagen remodeling to native, functional fascia-like tissue at the implant site.

## 1. Introduction

Different surgical meshes are commonly used in various applications throughout the body, such as treating burns, performing abdominal wall hernia repairs, and in breast procedures [1,2,3,4,5]. Commercially available biologic meshes provide a collagen-based scaffold for supporting host tissue deposition and remodeling [1,6,7]. As part of normal metabolic processes and tissue maintenance, or in response to injury, collagen is typically digested and replaced or remodeled [8]. Similarly, implanted biologic meshes integrate with surrounding host tissue through a series of events that can be classified into 3 stages of the wound healing response: inflammation, proliferation/resorption, and remodeling [8,9]. The inflammation stage is primarily characterized by the infiltration of immune cells into the implant site, activated by pro-inflammatory cytokines [2,8,9]. Although inflammation is critical for the wound healing response and mesh integration, excessive inflammation may lead to mesh degradation or encapsulation due to resulting severe fibrosis [10,11]. The proliferation stage is characterized by host tissue deposition [8], concomitant with biologic mesh collagen resorption. This new collagen synthesis contributes to the overall mechanical strength retention of the repair [8], which is essential for effective abdominal wall reconstruction [12]. The remodeling stage is characterized by fibroblast infiltration and neovascularization at the implant site, as well as implant-to-host collagen turnover.

There are many commercially available biologic mesh products, which differ in tissue of origin (e.g., type of species, anatomic location of harvest), as well as manufacturing processes, which may contribute to differences in overall kinetics of the host wound healing response and the mesh resorption profile [9,13,14]. Although limited, in vivo studies that evaluate changes in the implanted biologic mesh and surrounding host tissue over time periods >6 months are valuable for understanding biologic mesh integration and provide insight into successful clinical outcomes [7,12,14].

Strattice™ Firm, an electron-beam terminally sterilized porcine-derived acellular dermal matrix (E-PADM) commonly used for repair of hernia and other body wall defects [15], is available in different sizes and thicknesses to support a variety of surgical repairs requiring soft tissue support [16,17]. Strattice Firm is processed by a proprietary method that removes cellular components while retaining the biochemical and structural integrity of the matrix [15,18,19]. Unlike other biologic mesh products, Strattice Firm is not intentionally crosslinked, thus minimizing potential immune/inflammatory responses, while supporting tissue regeneration and reconstruction [2,7,18,19]. Viral reduction and electron-beam sterilization are applied to Strattice Firm [7,19]. Furthermore, the α-Gal epitope, which elicits xenogenic immune responses, is reduced through enzymatic cleavage during the manufacturing process [7,18,19]. A previous study has shown reduced amounts of IgG antibodies against the α,-Gal epitope without damage to its structural integrity [7].

Previous preclinical studies in a rodent subcutaneous implant model [20,21,22] and a primate abdominal wall repair model [7,19,22] have demonstrated that biologic meshes, including Strattice, retain their mechanical strength to the extent that their matrix proteins remain undamaged from processing. Furthermore, in these studies [7,19,20,21,22], Strattice showed biologic remodeling post-implantation commensurate with the surrounding host tissue. With the increasing number of commercially available biologic mesh materials, preclinical studies that evaluate host tissue integration and biologic remodeling in primates are clinically relevant because they show that these mesh materials exhibit similar immune and foreign body responses to those in humans [22]. The objective of the current study was to characterize collagen remodeling of Strattice Firm in a nonhuman primate model of abdominal wall repair by evaluating host biologic response kinetics during wound healing. The abdominal wall repair model has been used in previous studies [7,12,14,19,22,23] and represents the worst-case scenario with respect to tension loading as relates to healing and graft performance parameters.

## 2. Materials and Methods

### 2.1. Biomaterials

Strattice Firm (Allergan Aesthetics, an AbbVie Company, Branchburg, NJ, USA) samples, referred to as E-PADM throughout, approximately 8 × 8 cm, from 6 different lots were tested at each time point. Materials were prepared according to the manufacturer’s instructions for use prior to all testing procedures [15].

### 2.2. Study Design

E-PADM (*n* = 6 lots/time point) was implanted in a full-thickness abdominal wall bridging defect in African green monkeys (*N* = 24), as previously described [12,14]. Histological, immunohistochemical, and biochemical assessments were conducted at 1, 3, 6, and 9 months post-implantation. To assess inflammation, tissue cytokine assays and serum immunoglobulin G (IgG) enzyme-linked immunosorbent assays (ELISA) were conducted. To assess proliferation/resorption, immunohistochemistry (IHC) staining for matrix metalloproteinase-1 (MMP-1) and its inhibitor, tissue inhibitor matrix metalloproteinase-1 (TIMP-1), as well as histology scoring for E-PADM collagen resorption and host tissue deposition, were performed. Changes in E-PADM thickness over time were also evaluated. To assess remodeling, picrosirius red staining, as well as histologic scoring for fibroblast infiltration and neovascularization, were conducted. Changes in E-PADM thickness over time also were evaluated to assess E-PADM macrostructure and remodeling.

### 2.3. Animals

Twenty-four adult male African green monkeys (*Chlorocebus sabaeus*, *C. athiops saebeus*) weighing 5–9 kg (Worldwide Primates, St. Kitts, Eastern Caribbean) were quarantined for a minimum of 21 days and screened for general health before study entry. All animals were fed a standard primate diet twice per day. All animal procedures and care were performed at Allergan Aesthetics (Irvine, CA, USA) and were approved by the Allergan Institutional Animal Care and Use Committee (approval number 1986-D08-054-2019). Each animal was randomly implanted with 1 of 6 different lots of E-PADM within the abdominal wall, as previously described [7,12,14,19]. Six animals, representing 1 each of the 6 lots of E-PADM, were euthanized at 1, 3, 6, and 9 months for E-PADM explantation.

### 2.4. Primate Abdominal Wall Repair Model

This protocol has been described in previously published studies [7,12,14,19,22,23]. Animals were fasted for up to 16 h before the procedure. Animals were initially anesthetized by intramuscular (IM) injection of ketamine (5–10 mg/kg), with xylazine (0.6 mg/kg; Decrhra Veterinary Products, Overland Park, KS, USA). Animals were then intubated endotracheally and received 0.5–3% isoflurane for the duration of the procedure. Following anesthesia and surgical site preparation, a longitudinal mid-abdominal incision was made to expose an area of the abdominal muscle (≈3 × 7 cm), and a bilateral longitudinal full thickness defect was created by removal of fascia, rectus muscle, and peritoneum. Defects were repaired with a single piece of E-PADM that was trimmed to be equal in size to the defect (≈3 × 7 cm). The E-PADM was anchored at each of the 4 corners with a single interrupted polypropylene suture and secured to the edges of the rectus abdominis muscle and fascia in a continuous pattern also with polypropylene sutures. Subcutaneous tissues were closed with a continuous, absorbable monofilament polydioxanone (PDS-II) sutures (Ethicon, Raritan, NJ, USA), while the skin was closed with interrupted nylon sutures. Following surgery, subcutaneous (SC) injections of buprenorphine (0.2 mg/kg) were administered no more than every 72 h until no longer deemed necessary. Oral or SC injections of meloxicam (0.3 mg/kg) or a similar nonsteroidal anti-inflammatory drug were administered once daily for at least 3 days post-surgery. Euthanasia was performed by intravenous overdose of sodium pentobarbital at either 1, 3, 6, or 9 months post-implantation. Following euthanasia, the repair site was exposed, and the E-PADM and surrounding host tissue were excised by making an incision approximately 2–3 cm around the outside circumference of the E-PADM explantation site.

### 2.5. Tissue Cytokine Assay

A 1 × 1 cm sample taken from the E-PADM implant site from each animal was used to probe for human pro-inflammatory cytokines. As previously described [24], samples were flash-frozen in liquid nitrogen and stored at −80 °C. Prior to testing, explanted, flash-frozen samples (*N* = 30, *n* = 6 per time point) were then each incubated in culture media for a period of 1, 3, 5, 24, and 48 h to extract cytokines from tissue, and the extraction media were collected for further analysis.

The media samples were then diluted 1:2 using the VPLEX Proinflammatory Panel 1 Human Kit (Meso Scale Diagnostics, Rockville, MD, USA) to quantify the following cytokines: IL-1β, IL-2, IL-4, IL-6, IL-8, IL-10, IL-12p70, IL-13, IFN-γ, and TNF-α. Standard curves were run in duplicate using known concentrations of each cytokine analyte. The samples were assayed in triplicate, and individual readings from each incubation time were combined to calculate the total extracted cytokine concentrations for each tissue sample. Final concentrations were determined by correlating the sample signal intensities to the 4-parameter log-fit standard curve for the respective analytes using Meso Scale Diagnostics Discovery Workbench version 4.0 software. Final data were reported as pg of cytokine per mg of extracted tissue.

### 2.6. Serum IgG Antibody ELISA

Serum samples were collected from each animal at time 0, 1, 2, 4, 12, 24, and 36 weeks post-implantation. An ELISA assay was performed by incubating serially diluted serum in 96-well plates that had been coated with lot-matched, non-implanted E-PADM material, washed in 50 mL of saline, freeze-dried, and cryo-milled to form a fine powder. Alkaline phosphatase linked to anti-human IgG (γ chain specific; Sigma, St. Louis, MO, USA) was used as the secondary antibody to primary E-PADM-specific antibodies formed in serum and bound to E-PADM-coated wells. P-Nitrophenyl phosphate buffer was used as a substrate for alkaline phosphatase, and the absorbance of the samples was read spectrophotometrically at 405 nm. Optical density readings from secondary antibody-only control wells were subtracted from all results. Final data were reported as mean fold increase over time 0 or baseline.

### 2.7. Histology/Immunohistochemistry

Explanted E-PADM samples were collected at 1, 3, 6, and 9 months post-implantation, placed in 10% v/v neutral buffered formalin, embedded in paraffin blocks, and sectioned onto glass slides. For collagenase enzyme quantification, slides were stained with anti-MMP-1 and anti-TIMP-1 monoclonal antibodies (Fitzgerald Industries International, Acton, MA, USA). To assess general inflammation, inflammatory cell types, E-PADM collagen resorption, host tissue deposition, E-PADM site fibroblast infiltration, and E-PADM site neovascularization, slides were stained separately using Masson’s Trichrome and hematoxylin and eosin (H&E). To assess the thickness of collagen fibers at the E-PADM site, slides were stained using picrosirius red, which indicates collagen remodeling [25], visualized using polarized light microscopy, and evaluated. A total of 231 slides were scored by an independent, blinded, board-certified veterinary histopathologist, including 1 pre-implant sample from each of the 6 lots used. Histologic scoring criteria are shown in Table 1.

#### 2.7.1. MMP-1 and TIMP-1 IHC Image Analysis

As previously described [23], the implanted tissue area for each prepared immuno-stained slide was identified and demarcated as the region of interest (ROI); staining artifacts (e.g., folds, tears) were excluded from ROIs. The immuno-positive area within each ROI was measured, compared to the total ROI area, and reported as a percentage (% immuno-positive area = (immuno-positive area within ROI [µm^2^]/total ROI area [µm^2^]) × 100). Slides were scanned using the Aperio AT2 whole slide scanner (Leica Biosystems, Wetzlar, Germany), and image analysis was conducted using Visiopharm software (Hørsholm, Denmark) by Inotiv (West Lafayette, IN, USA).

#### 2.7.2. Implant Site Morphometry

Standard Masson’s trichrome–stained histologic sections were used to measure implant site thickness, which was defined as the thickness of any E-PADM present in addition to any host tissue deposition. T = 0 (un-implanted E-PADM) was compared to samples explanted at 1, 3, 6, and 9 months. A total of 5 equally spaced thickness measurements were made across the length of each sample and then averaged.

### 2.8. Statistical Analyses

Data from tissue cytokine assays, serum IgG antibody ELISA, as well as IHC image analysis and morphometry were summarized using descriptive statistics. Histology scores were analyzed using the non-parametric Kruskal–Wallis Test individually for each parameter. Pairwise group comparisons were conducted to compare histology scores from 1 month to scores at later time points. Morphometry data were analyzed using a 2-sample *t* test. Statistical analyses were performed using Minitab (State College, PA, USA).

## 3. Results

### 3.1. Tissue Cytokine Assay

**Figure 1:**
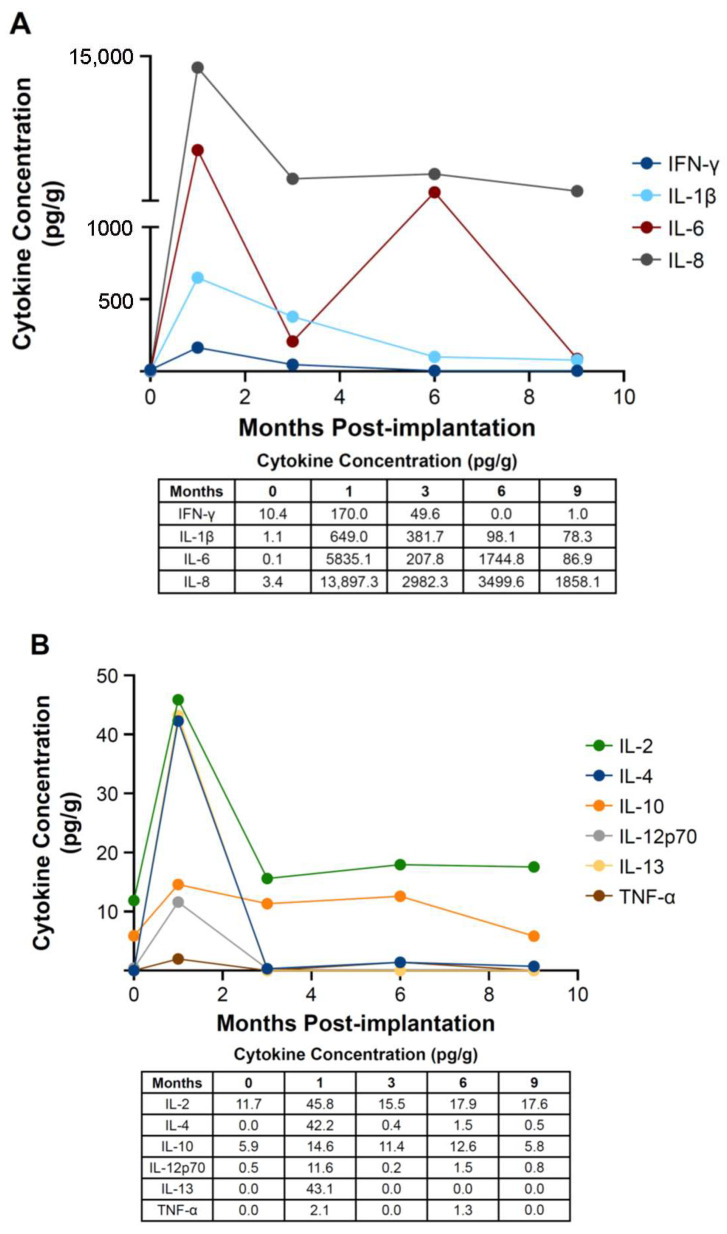
Key pro-inflammatory tissue cytokines (IFN-γ, IL-1β, IL-6, and IL-8) peaked 1 month post-implantation and subsided to baseline by 6 months. Total concentration (pg/g) over time for (**A**) cytokines > 100 pg/g (IFN-γ, IL-1β, IL-6, and IL-8) and (**B**) cytokines < 100 pg/g (IL-2, IL-4, IL-10, IL-12p70, IL-13, and TNF-α). *n* = 6 animals/time point. IL, interleukin; IFN, interferon; TNF, tumor necrosis factor.

Several pro-inflammatory tissue cytokines, including IFN-γ, IL-1β, IL-6, and IL-8, peaked at 1 month post-implantation, reaching concentrations of 170.0 pc/g, 649.0 pg/g, 5835.1 pg/g, and 13,897.3 pg/g, respectively (Figure 1A). Increases in other cytokines, including IL-2, IL-4, IL-10, IL-12p70, IL-13, and TNF-α, also were detected, although the increases were not substantial, with concentrations below 100 pg/g, at 1 month post-implantation (Figure 1B). Most cytokine concentrations either plateaued or subsided to near-baseline levels (pre-implantation) by 3 to 9 months.

### 3.2. Serum IgG ELISA

**Figure 2:**
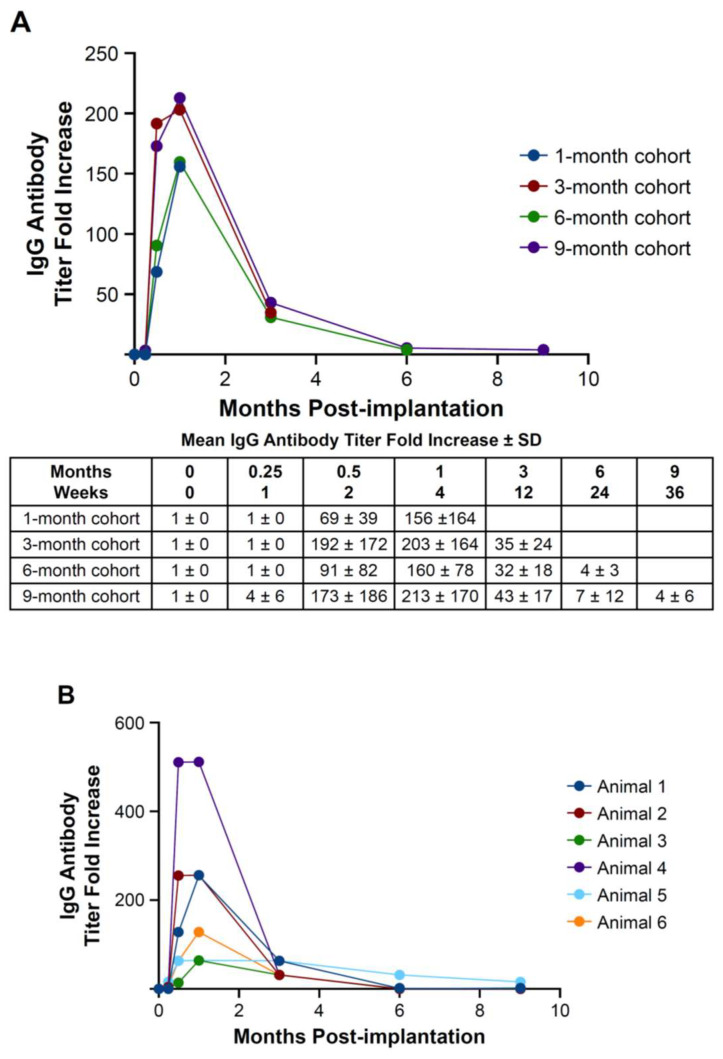
E-PADM-specific serum IgG antibody titer peaked 1 month post-implantation and subsided to baseline by 6 months. (**A**) Mean IgG titer fold increase ± SD for all animal cohorts and (**B**) 9-month cohort only. The individual curves represent each sample (animal number) in the 9-month cohort. *n* = 6 animals/time point. E-PADM, E-beam-sterilized porcine-derived acellular dermal matrix; IgG, immunoglobulin G; SD, standard deviation.

E-PADM-specific serum IgG antibody titer peaked 1 month post-implantation with an average 183-fold increase from baseline (range, 156–213-fold increase across all cohorts; Figure 2A,B). At 3 months post-implantation, IgG antibody titer decreased to an average 36.4-fold difference from baseline (range, 32–43), then subsided to <10-fold difference from baseline by 6 and 9 months.

### 3.3. MMP-1 and TIMP-1 IHC Image Analysis

**Figure 3:**
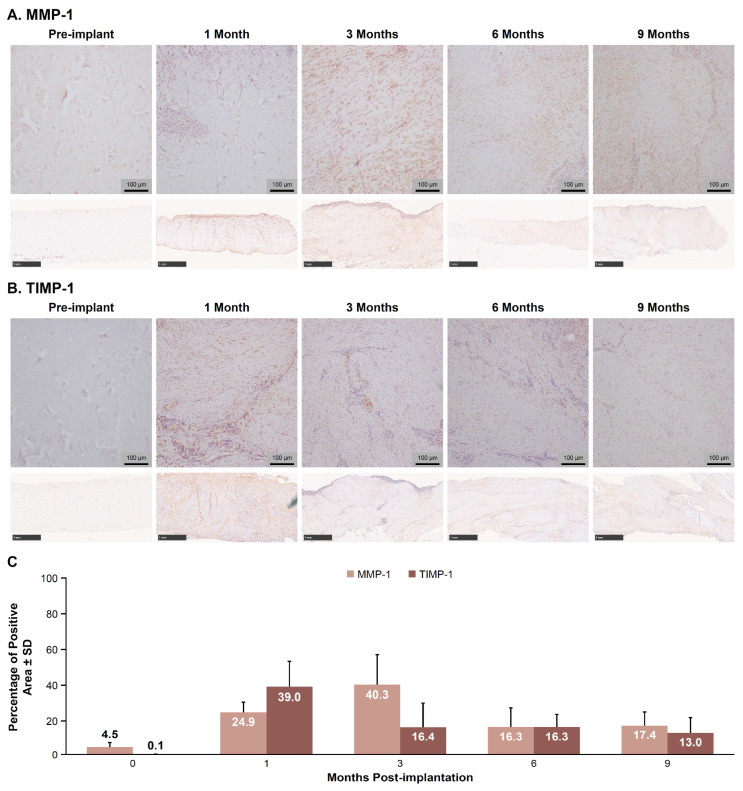
MMP-1-positive tissue area peaked at 3 months post-implantation, then decreased and stabilized by 6 months, while TIMP-1-positive tissue area peaked at 1 month post-implantation, then decreased and stabilized by 9 months. Representative high- (100×; scale bars, 100 µm) and low-(10×; scale bars, 1 mm) magnification images of (**A**) MMP-1 and (**B**) TIMP-1 from sample tissues collected pre-implantation and 1, 3, 6, and 9 months post-implantation. There was a higher abundance of MMP-1- and TIMP-1-positive tissue area at 3 months and 1 month post-implantation, respectively, compared with pre-implantation. (**C**) Mean percentage ± SD of MMP-1- and TIMP-1-positive areas. *n* = 6 animals/time point. MMP-1, matrix metalloproteinase 1; SD, standard deviation; TIMP-1, tissue inhibitor matrix metalloproteinase 1.

Representative images of MMP-1 and TIMP-1 IHC staining are shown in Figure 3A and Figure 3B, respectively. The mean percentage (±SD) of MMP-1-positive tissue area peaked at 40.3 ± 16.9% at 3 months, declined to 16.3 ± 11.1% by 6 months, and remained steady at 17.4 ± 7.6% by 9 months (Figure 3C). The mean percentage of TIMP-1-positive tissue peaked at 39.0 ± 14.3% at 1 month, declined to 16.4 ± 13.5% by 3 months, and remained steady at 13.0 ± 8.8% by 9 months (Figure 3C).

### 3.4. Histology Scoring

**Figure 4:**
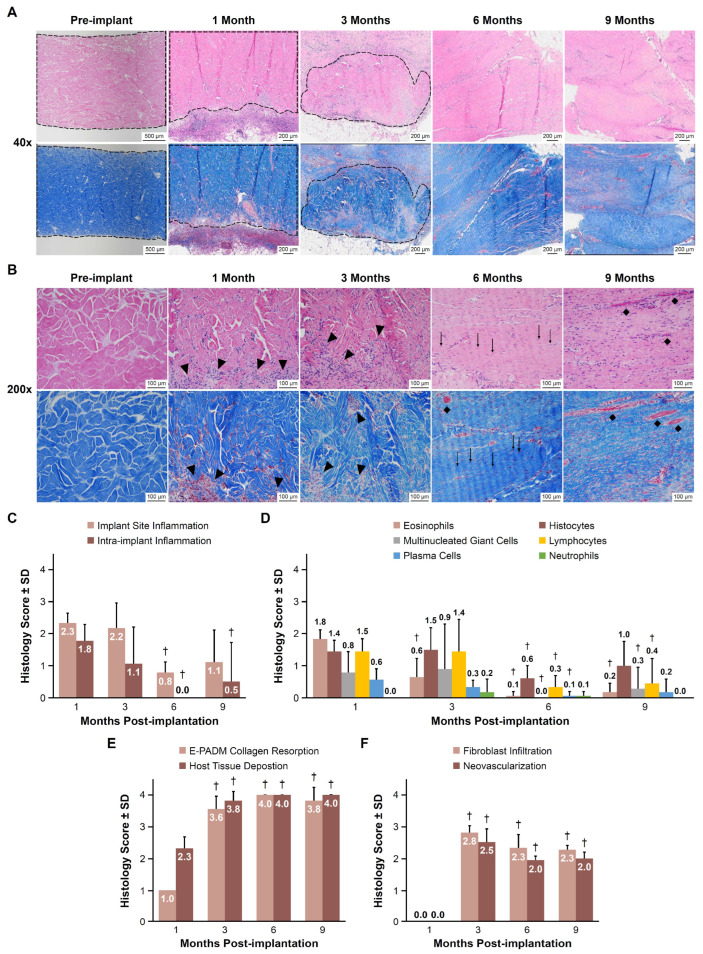
Representative hematoxylin and eosin–stained and Masson’s trichrome images showed decreasing levels of visible inflammatory cells and the transition of E-PADM-associated collagen to infiltrated host tissue from pre-implantation to 9 months. Pre-implantation images show complete cell removal after the decellularization process during the manufacturing of E-PADM. (**A**) Representative images taken at 40× magnification differentiate between the E-PADM and the area surrounding the implant. The dotted line indicates the transition of E-PADM-associated collagen in pre-implanted E-PADM 1- and 3-month post-implantation samples to infiltrated host tissue at 6- and 9-month post-implantation samples. (**B**) Representative images taken at 200× magnification show the E-PADM implant undergoing these changes. Panel B shows inflammatory cells (arrowheads), vessels (diamonds), and fibroblast cells (arrows) on fascia-like collagen fibers, which indicate collagen remodeling post-implantation. Scale bars range from 100 µm to 500 µm and are indicated on the corresponding images. Mean histology scores ±SD for (**C**) general inflammation, (**D**) inflammatory cell types, (**E**) E-PADM collagen resorption and host tissue deposition over time, and (**F**) fibroblast infiltration and neovascularization over time. *n* = 6 animals/time point. A representative histological section used for host response scoring of the E-PADM is shown in Figure S1, and the scoring criteria are elaborated in Table 1. E-PADM, electron-beam terminally sterilized porcine-derived acellular dermal matrix; SD, standard deviation. † *p* < 0.05 versus 1 month.

Representative H&E-stained images showed decreasing levels of visible inflammatory cells and the transition of E-PADM-associated collagen to primate fibroblast and vessel-infiltrated host tissue from 1 to 9 months (Figure 4A,B). The degree of implant site inflammation (areas external to the E-PADM boundaries), as indicated by the assigned histologic score, was considered mild at 1 and 3 months (2.3 ± 0.3 and 2.2 ± 0.8, respectively), then significantly decreased to minimal at 0.8 ± 0.3 at 6 months (*p* < 0.01; Figure 4C). Intra-implant inflammation (areas within the E-PADM implant) was also considered mild at 1.8 ± 0.5 at 1 month, then significantly decreased to absent or minimal at 6 and 9 months (0.0 ± 0.0 and 0.5 ± 1.2, respectively; *p* < 0.05; Figure 4C). Observed levels of specific inflammatory cells were mild at 1 month and were predominantly eosinophils, histiocytes, and lymphocytes (Figure 4D). Inflammatory cells continuously decreased to absent or minimal levels at 9 months.

**Figure 5:**
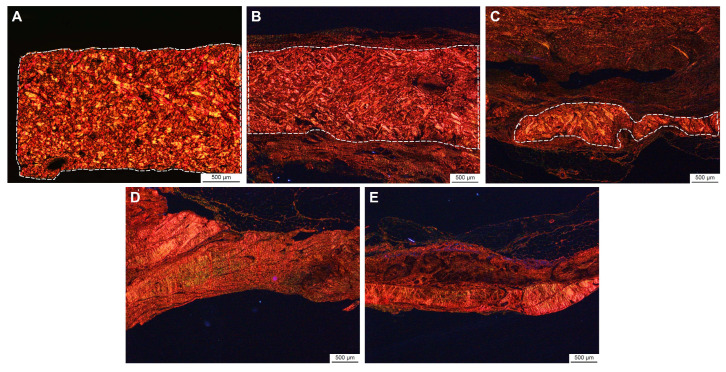
Representative images of picrosirius red staining taken at 20× magnification (scale bar, 500 µm) demonstrates the replacement of E-PADM collagen that has reticular-like collagen fibers at pre-implantation with host-associated neo-collagen that has fascia-like collagen fibers by 6 months post-implantation. (**A**) E-PADM prior to implantation with a birefringent lattice-like dermal structure. (**B**) E-PADM 1 month post-implantation with birefringent dermal structure surrounded by nonbirefringent host primate tissue. A higher-magnification image taken at 40× is shown in Figure S2. (**C**) E-PADM 3 months post-implantation with the majority of dermal tissue remodeled to nonbirefringent host primate tissue. (**D**) Six-month and (**E**) 9-month explanted E-PADMs with no evidence of birefringent dermal structure. Dotted lines demarcate the remaining E-PADM from the surrounding tissue. 20× magnification. E-PADM, electron-beam terminally sterilized porcine-derived acellular dermal matrix.

E-PADM collagen resorption significantly increased between 1 and 3 months (1.0 ± 0.0 vs. 3.6 ± 0.4, respectively, *p* < 0.01; Figure 4E), with E-PADM being difficult to distinguish from surrounding, remodeled host tissue. Collagen resorption of E-PADM collagen continued at a slower rate through 6 and 9 months (4.0 ± 0.0 and 3.8 ± 0.4, respectively, *p* < 0.01 vs. 1 month), with negligible evidence of the original E-PADM implant dermal collagen with reticular-like structure remaining, as it was fully replaced by host collagen with fascia-like fibers. Concomitantly, host tissue deposition significantly increased between 1 and 3 months (mean histology scores [SD] of 2.3 ± 0.4 vs. 3.8 ± 0.3, respectively, *p* < 0.01), with host tissue observed throughout the implant, (Figure 4F) and stabilized by 6 and 9 months (4.0 ± 0.0 for both 6- and 9-month time points, *p* < 0.01 vs. 1 month), with diffuse host tissue expansion throughout the E-PADM, as demonstrated by H&E staining. Additional staining of E-PADM with picrosirius red, both prior to and following implantation, revealed a gradual transition from primarily red-staining, mature, reticular-like, birefringent dermal collagen at pre-implantation, to yellowish-green staining neo-collagen with fascia-like fibers following implantation (Figure 5).

E-PADM site fibroblast infiltration also increased significantly between 1 and 3 months (0.0 vs. 2.8 ± 0.2, respectively, *p* < 0.01), then somewhat decreased, reaching equilibrium by 6 and 9 months (2.3 ± 0.4 and 2.3 ± 0.1, respectively, *p* < 0.01 vs. 1 month; Figure 4D). Similar results were observed for E-PADM site neovascularization, with a significant increase in the presence of vessels between 1 and 3 months (0.0 vs. 2.5 ± 0.4, respectively, *p* < 0.01), followed by a slight decrease at 6 months and maintenance of the same degree of vascularization at 9 months (2.0 ± 0.1 and 2.0 ± 0.2, respectively, *p* < 0.01 vs. 1 month; Figure 4D).

### 3.5. Implant Site Morphometry

**Figure 6:**
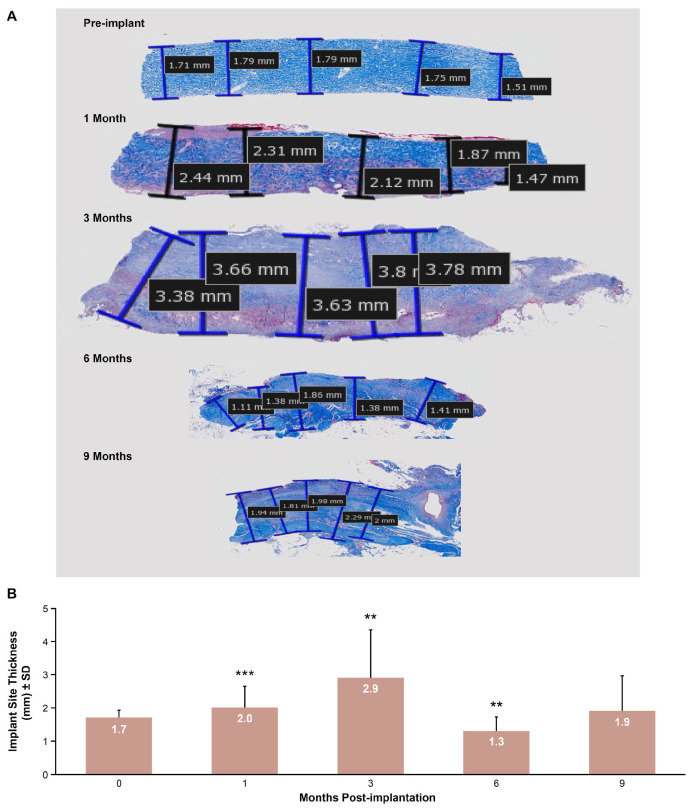
(**A**) Representative images taken at 10× magnification showing E-PADM thickness measurements at baseline (pre-implantation, month 0) and 1, 3, 6, and 9 months post-implantation. (**B**) Mean E-PADM implant site thickness (mm ± SD) increased up to 3 months, followed by decrease and stabilization at 6 and 9 months, respectively. *n* = 6 animals/time point. E-PADM, electron-beam terminally sterilized porcine-derived acellular dermal matrix. SD, standard deviation. ** *p* < 0.01 versus month 0 (pre-implantation); *** *p* < 0.001 versus month 0 (pre-implantation).

Representative H&E-stained images showed increased thickness from pre-implantation to 3 months, before returning to baseline levels by 9 months (Figure 6A). Mean implant thickness (±SD) significantly increased over time, from 1.7 ± 0.2 mm at pre-implantation to a maximum of 2.9 ± 1.5 mm at 3 months (*p* < 0.01), before returning to pre-implantation thickness levels of 1.9 ± 1.1 mm by 9 months (*p* = 0.06; Figure 6B).

## 4. Discussion

Various tissue sources (e.g., species, anatomic locations) and manufacturing processes may contribute to differences in the unique resorption profile of biologic meshes [9,13,14]. Studies that evaluate long-term in vivo remodeling of biologic meshes, especially in nonhuman primate models, are increasingly valuable to clinicians for application-specific mesh selection guidance, which may ultimately affect clinical performance and postoperative outcomes [7,12,14].

The current study examined collagen remodeling of E-PADM in an Old World primate model, which has been shown to be clinically relevant for evaluation of the biological and immunological response to implanted graft materials [7,14]. These models generally allow investigators to establish the mechanisms of action of implanted tissue graft materials without the confounding factors typically associated with xenogenic transplantation [12,14,19,26]. Furthermore, primate models, such as the African green monkey (*Chlorocebus sabaeus*, *C aethiops sabaeus*), have an established immunologic homology to humans and are historically suitable for differentiating among decellularized matrices, whether derived from allogenic or xenogenic origins [12,14,19,26].

Previous nonhuman primate studies that assessed the use of a surgical mesh to repair bridging defects in nonhuman primates have demonstrated revascularization, cellular repopulation, and the absence of significant immune response with biologic mesh [7,12,14,19,22]. The full-thickness abdominal wall repair represents a demanding healing model because implanted materials are exposed to the intrinsic forces of the abdominal wall space, which have been shown to impact overall collagen remodeling in this indication [12,14,19]. Edge-to-edge apposition of the tissues also creates a challenging scenario to ensure durable repair. The results from this preclinical study should therefore be considered to represent the worst-case scenario with respect to graft performance parameters relating to the remodeling of E-PADM.

Implantation of E-PADM contributed to induction of a wound healing response, which was the primary driver of E-PADM collagen remodeling. The presence of E-PADM elicited a mild inflammatory phase, characterized by a transitory increase in local tissue cytokine levels, (Figure 1), systemic serum IgG (Figure 2), and specific inflammatory cells (Figure 4D), lasting 1 to 3 months. The overall elevation in pro-inflammatory cytokine levels was predominated by IL-6 and IL-8, which are major regulators of the acute inflammatory response and play an important role in the transition from the inflammatory to proliferative stage of wound healing [27,28,29] (Figure 1). The transitory increase in serum IgG and tissue cytokines is important for the timely resolution of wound healing [27]. The infiltration of host cells (Figure 4F) and newly deposited collagen (Figure 4E) within E-PADM peaked between 3 and 6 months post-implantation and coincided with ≈75% to 100% E-PADM collagen resorption during this same time period (Figure 4E). During the final remodeling stage of the wound healing response, E-PADM transitioned from a dense, reticular porcine dermal collagen structure to a more loosely organized host collagen structure, then finally to a mature and lamellar host collagen morphology in the 3 to 6 month timeframe. This morphology has a fascial-like architecture, which is similar to surrounding native tissues at the site of implantation (Figure 5). Furthermore, MMP-1 and TIMP-1 expression levels (Figure 3C) and implant thickness data (Figure 6) demonstrated that biochemical changes coincided with observed macrostructural changes, with MMP-1 and implant site thickness reaching peak levels at 3 months, concomitant with the height of remodeling, followed by a decrease in MMP-1 and implant site thickness to pre-implant thickness levels by 6 months. These changes in implant thickness support the use of E-PADM as a scaffold for the synthesis of new collagen, thus supporting remodeling [7,20]. A previous study also showed that the Strattice Firm retains its thickness in a rodent subcutaneous implant model [21].

Functional remodeling of collagen-based surgical meshes requires a delicate balance between the degradation of collagen by matrix metalloproteinases and the synthesis of new collagen by fibroblasts [8]. This process continues even months following primary wound repair [8]. The results of the current study suggest that retention of the native structure and biochemistry of E-PADM during the manufacturing process may contribute towards successful host tissue acceptance and integration [13,14,19,30]. In contrast, other types of biologic meshes consisting of damaged or cross-linked collagenous matrices either elicit excessive inflammation and become rapidly resorbed or do not allow host cell infiltration and the formation of new vessels within the matrix even after 6 months post-implantation [14,19].

The current study complements previously published studies, which evaluated the host biologic response of implanted E-PADM in rodents [20], porcine [31], and primate models [19]. While not conducted in the current study, previous studies have described the mechanical strength of E-PADM, out-of-package, in vitro using collagenase tensile testing assays, and in vivo using rodent and primate implantation models [7,22]. Unlike previous studies, which had shorter time frames (e.g., up to 6 months), this study is the first to evaluate longer-term kinetics (9 months) of remodeling in a nonhuman primate model.

The breadth of knowledge in the prosthetic-based abdominal wall repair space is vast, further developing over the past several decades since publication of the seminal clinical study positively that supported the use of surgical mesh over primary repair alone [32]. Compared with suture repair alone, mesh repair had significantly fewer incidences of recurrences and postoperative infection [32]. However, subsequent studies have shown that the type of surgical mesh (e.g., synthetic, biologic, or biosynthetic) affects the incidence of recurrences and postoperative complications [33,34]. Previous studies in nonhuman primate models have revealed different mechanisms of action and outcomes for synthetic, biological, and biosynthetic meshes [7,12,14,19,22,35]. In the current study, we further elucidated the mechanism of action of regenerative remodeling for E-PADM by analyzing specific markers of local and systemic inflammatory responses, as well as markers of collagen remodeling, in a well-established nonhuman primate model. The study results expand on the current understanding of E-PADM biology and its ability to remodel into functional supportive tissue due to its retained matrix integrity rather than to elicit a negative recognition or foreign body response, as is typically observed for denatured or cross-linked biologic materials or synthetic materials [7,12].

A limitation of this study is the use of a bridging defect model in nonhuman primates. Although not clinically relevant in terms of current abdominal wall repair technique, this model serves as a worst-case scenario for wound healing when subjected to the forces of the dynamic abdominal wall environment. Additional studies in animal models that closely replicate current clinical practice for mesh placements (i.e., overlay, underlay, and retrorectus placement techniques) are warranted [36]. Future studies should also consider building upon existing research and expanding on prior comparisons with other materials (collagen-based/synthetics/hybrid materials) [7,12,14,19,22,35]. Furthermore, specific cell types/lineages and signaling pathways associated with each stage of the wound healing response and long durations of implantation (≥12 months) can be examined.

## 5. Conclusions

Implantation of E-PADM in a nonhuman primate abdominal wall repair model contributed to the induction of a wound healing response, which was the primary driver of E-PADM collagen remodeling. The current study indicates that, in the context of normal wound healing, the implantation of E-PADM elicited a mild inflammatory response phase lasting 1–3 months, with a period of matrix-to-host collagen turnover and remodeling that reached equilibrium by 3–6 months. Collagen remodeling resulted in a transition to host tissue histologically resembling the surrounding implantation site and having a thickness similar to E-PADM at the time of implantation.

## Figures and Tables

**Table 1:** Histopathology scoring criteria.

Evaluation	Scoring Criteria
Inflammation/inflammatory cells	0 = Absent 1 = Rare, minimal, 1–5/per high-power field (40×) 2 = Mild, 5–10/per high-power field (40×) 3 = Heavy infiltrate with preservation of local architecture 4 = Packed, with effacement of regional architecture
Collagen resorption	0 = Original E-PADM intact, borders clearly demarcated (0% resorbed) 1 = E-PADM minimally resorbed (<25%), with some separation by host tissue/infiltrates 2 = E-PADM notably resorbed (≈25–75%), difficult to distinguish scaffold from host tissue 3 = E-PADM markedly resorbed (≈>75%), difficult to distinguish scaffold from host tissue 4 = No evidence of E-PADM remaining, ≈100% resorbed
Host tissue deposition	0 = Absent 1 = Host tissue restricted to periphery of E-PADM 2 = Host tissue present within the E-PADM interstitium, not extending to center 3 = Host tissue; present throughout E-PADM, including center 4 = Host tissue diffusely expands throughout E-PADM
Fibroblast infiltration	0 = Absent (i.e., fibrocytes present but no fibroblasts, typical of quiescent native tissue) 1 = Minimal, rare fibroblasts present within connective tissue 2 = Mild, multifocal presence, fibroblasts constitute minority of connective tissue 3 = Moderate, diffuse presence, fibroblasts are notable component of connective tissue 4 = Marked, diffuse presence, fibroblasts predominate connective tissue response
Neovascularization	0 = Absent 1 = Minimal capillary proliferation, focal, 1–3 buds/area 2 = Clusters of 4–7 capillaries with supporting fibroblastic structures 3 = Broad band of capillaries, or larger vessels (arteries/veins) with supporting structures 4 = Extensive bands of vessels with supporting fibroblastic structures

## Data Availability

AbbVie is committed to responsible data sharing regarding the clinical trials we sponsor. This includes access to anonymized, individual, and trial-level data (analysis data sets), as well as other information (e.g., protocols, clinical study reports, or analysis plans), as long as the trials are not part of an ongoing or planned regulatory submission. This includes requests for clinical trial data for unlicensed products and indications. These clinical trial data can be requested by any qualified researchers who engage in rigorous, independent, scientific research, and will be provided following review and approval of a research proposal, Statistical Analysis Plan (SAP), and execution of a Data Sharing Agreement (DSA). Data requests can be submitted at any time after approval in the US and Europe and after acceptance of this manuscript for publication. The data will be accessible for 12 months, with possible extensions considered. For more information on the process or to submit a request, visit the following link: https://vivli.org/ourmember/abbvie/ then select “Home”.

## Supplementary Materials

The following supporting information can be downloaded at: https://www.mdpi.com/article/10.3390/bioengineering12080796/s1, Figure S1: Representative hematoxylin and eosin–stained section used for host response scoring of E-PADM; Figure S2: A higher-magnification picrosirius red image of E-PADM at 1 month post-implantation with birefringent dermal structure surrounded by nonbirefringent host primate tissue.

## Abbreviations

The following abbreviations are used in this manuscript:

ELISA	enzyme-linked immunosorbent assay
E-PADM	electron-beam terminally sterilized porcine-derived acellular dermal matrix
IgG	immunoglobulin G
IHC	immunohistochemistry
IM	intramuscular
