# Supplementary material for: Collagen Remodeling of Strattice™ Firm in a Nonhuman Primate Model of Abdominal Wall Repair
Source: Bioengineering (Basel). 2025 Jul 24;12(8):796. doi: 10.3390/bioengineering12080796 (PMC12383725; doi:10.3390/bioengineering12080796)

Supplementary Materials

Figure S1. Representative hematoxylin and eosin–stained section used for host response scoring of E-PADM (10×; scale bars, 500 µm). The heavy dashed line represents the original area of implanted E-PADM that was evaluated for inflammation, the presence of inflammatory cells, collagen resorption, host tissue deposition, fibroblast infiltration, and neovascularization. Asterisks (*) represent the remaining visible E-PADM structure. The lighter dotted line demarcates host tissue, inclusive of original host tissue as well as host tissue ingrowth into the E-PADM implant, represented by plus signs (+). E-PADM, electron-beam terminally sterilized porcine-derived acellular dermal matrix.

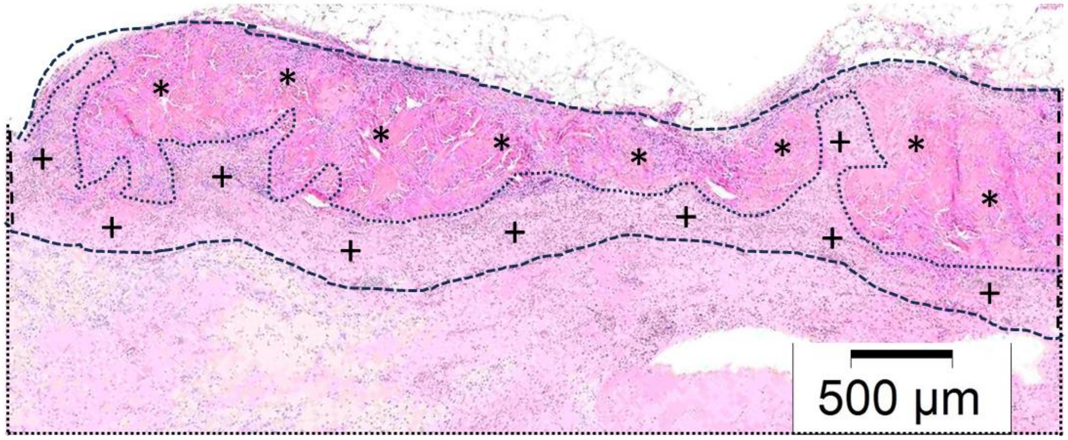

Figure S2. A higher-magnification picrosirius red image of E-PADM at 1 month post-implantation with birefringent dermal structure surrounded by nonbirefringent host primate tissue (40×; scale bars, 250 μm). E-PADM, electron-beam terminally sterilized porcine-derived acellular dermal matrix.

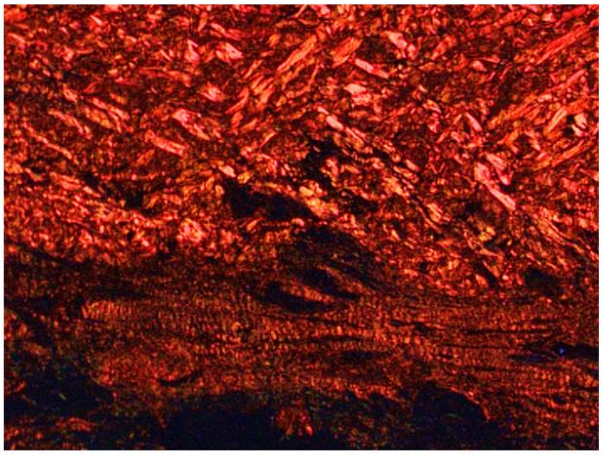

Supplement: Supplementary file 1 [file bioengineering-12-00796-s001.zip › bioengineering-3536568-supplementary.pdf]
